# Quantum Computing in Community Detection for Anti-Fraud Applications

**DOI:** 10.3390/e26121026

**Published:** 2024-11-27

**Authors:** Yanbo (Justin) Wang, Xuan Yang, Chao Ju, Yue Zhang, Jun Zhang, Qi Xu, Yiduo Wang, Xinkai Gao, Xiaofeng Cao, Yin Ma, Jie Wu

**Affiliations:** 1 Longying Zhida (Beijing) Technology Co., Ltd., Beijing 100020, China; wangyanbo@lyzdfintech.com (Y.W.); yangxuan@lyzdfintech.com (X.Y.); zhangyue@lyzdfintech.com (Y.Z.); zhangjun@lyzdfintech.com (J.Z.); xuqi@lyzdfintech.com (Q.X.); wangyiduo@lyzdfintech.com (Y.W.); gaoxinkai@lyzdfintech.com (X.G.); caoxiaofeng@lyzdfintech.com (X.C.); 2 Beijing QBoson Quantum Technology Co., Ltd., Beijing 100015, China; juc@boseq.com (C.J.); may@boseq.com (Y.M.)

**Keywords:** coherent ising machine (CIM), quantum computing, community detection, quadratic unconstrained binary optimization (QUBO), Louvain, simulated annealing, anti-fraud

## Abstract

Fraud detection within transaction data is crucial for maintaining financial security, especially in the era of big data. This paper introduces a novel fraud detection method that utilizes quantum computing to implement community detection in transaction networks. We model transaction data as an undirected graph, where nodes represent accounts and edges indicate transactions between them. A modularity function is defined to measure the community structure of the graph. By optimizing this function through the Quadratic Unconstrained Binary Optimization (QUBO) model, we identify the optimal community structure, which is then used to assess the fraud risk within each community. Using a Coherent Ising Machine (CIM) to solve the QUBO model, we successfully divide 308 nodes into four communities. We find that the CIM computes faster than the classical Louvain and simulated annealing (SA) algorithms. Moreover, the CIM achieves better community structure than Louvain and SA as quantified by the modularity function. The structure also unambiguously identifies a high-risk community, which contains almost 70% of all the fraudulent accounts, demonstrating the practical utility of the method for banks’ anti-fraud business.

## 1. Introduction

### 1.1. Quantum Computing for Anti-Fraud Applications

In modern society, fraud has become a serious threat to the financial security of individuals and businesses, resulting in billions of dollars of financial losses worldwide every year. This not only causes economic losses to the victims but also has an immeasurable impact on the social credit system. Among all sectors, the financial industry, due to its concentration of funds, has become a major target for fraudulent activities. Effective anti-fraud measures call for not only efficient algorithms for uncovering the hidden organizations among the crime units but also rapid implementation of the algorithms enabled by sufficient computational resources. The application of quantum computing in community detection algorithms is ideal for fulfilling this requirement.

Quantum computing (QC) is a rapidly growing research field that promises a novel paradigm to solve challenging computational problems. It was first introduced in the early 1980s by physicist Paul Benioff [1,2] and independently by Feynman [3]. Quantum computers utilize quantum bits (qubits) as the fundamental units of information storage [4], which can exist in superposition states of both |1〉 and |0〉, enabling them to hold exponentially more information compared to traditional computers. It has been argued that quantum computers could offer advantages in addressing specific problems such as NP-hard combinatorial optimization, often described as the superiority of quantum computing. There are many different paradigms and hardware implementations that can be used to build quantum computers. The two leading paradigms are gate-based QC [5] and adiabatic quantum computation (AQC) [6]. However, gate-based QC requires stringent environment control and sophisticated error correction, while AQC faces the main obstacle of improving the connection density between qubits, which will affect the efficiency of problem solving [7].

Recently, there have been some applications of quantum computing in fraud detection, mainly focusing on quantum algorithms. For example, Schuman et al. explored the use of Quantum Boltzmann Machines for unsupervised anomaly detection in fraud scenarios [8]. Bikku et al. [9] proposed a novel Quantum Neural Network (QNN) model that originated from the principles of the social learning theory to detect fraudulent reviews. Innan et al. presented a novel approach employing Quantum Graph Neural Networks (QGNNs) for financial fraud detection [10]. A comprehensive study examining the application of quantum algorithms to combat financial crimes was provided by Weinberg et al. [11]. Despite these latest advances in algorithms, no one has addressed the hardware challenges in applying quantum computing to fraud detection.

A Coherent Ising Machine (CIM) is a quantum computer developed according to optical parametric oscillation and spontaneous symmetry breaking principle [12,13,14,15,16,17,18] which can work at room temperature and solve large-scale problems, such as compression sensor problems [19] and polyhedron problems [20]. CIM searches for the ground state energy of an Ising model by using the phases of laser pulses to represent the spin directions of spins in the Ising model. The resulting phase configuration corresponds to the optimal solution of the problem equivalent to the Ising model. CIM has been used to solve optimization problems in various industrial sectors [21,22,23] and academic disciplines [24]. Current CIM research focuses on optimizing measurement feedback, pump rate and quantum state squeezing to improve the time-to-solution and success rate of solutions.

This study focuses on fraud detection in commercial banks and aims to construct a graph network structure. It seeks to employ community detection techniques implemented by CIM to effectively identify groups of accounts exhibiting fraudulent behavior so as to provide robust support for subsequent business decisions regarding the fraud risk of accounts within these communities.

### 1.2. Community Detection Algorithms

While AI-based supervised fraud detection methods abound, there are also many unsupervised techniques for detecting fraud. One such approach is clustering, which helps identify similarities among data samples and can reveal potential fraud features. In this context, we will focus on another unsupervised approach—community detection—which uncovers hidden community structures within a network for fraud detection.

Community detection is a fundamental task in network analysis aimed at uncovering the underlying structure of networks by identifying groups of nodes, known as communities, that are more densely connected internally than with the rest of the network. Over the years, numerous algorithms have been developed to tackle this problem, each with its strengths and limitations, reflecting the diverse nature of networks and the varying objectives of community detection.

One of the most widely used algorithms is the Louvain algorithm, which operates by optimizing modularity—a measure that quantifies the density of connections within communities relative to connections between communities. The Louvain method is particularly valued for its efficiency and scalability, making it suitable for large networks. However, although efficient, it has some notable drawbacks. For example, it is sensitive to initial conditions, such as the starting partition of the network, which leads to variability in the detected communities. Moreover, it is heuristic-based, meaning it may yield different results on different runs, even with the same input data, because the algorithm’s outcome can depend on the order in which nodes are processed.

The QUBO (Quadratic Unconstrained Binary Optimization) model for maximizing modularity can be considered another approach to community detection. In this context, the QUBO model is used to formulate the problem of modularity maximization as an optimization problem, where each binary variable indicates whether a node belongs to a particular community. The objective function is then formulated in such a way that its maximization corresponds to maximizing modularity. The QUBO model is flexible and can be adapted to different forms of modularity and constraints, allowing for customization based on specific requirements. Various optimization techniques can be employed to solve the QUBO model, such as simulated annealing (SA). In particular, the QUBO formulation is particularly well suited for quantum computing approaches, which can explore large solution spaces efficiently. In this paper, we solve the QUBO model by finding the ground state of its corresponding Ising model using Coherent Ising Machine (CIM).

### 1.3. The QUBO Model for Community Detection

**Figure 1:**
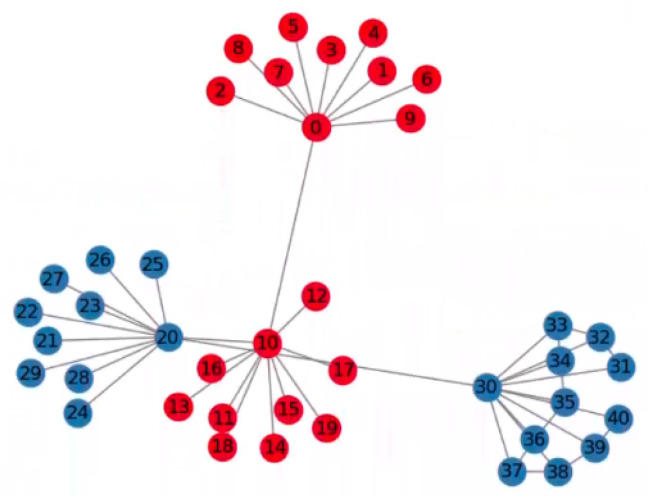
A graph illustrating the concept of modularity, showing a community structure in which the connections within a community are significantly more numerous than those between communities, i.e., with high modularity.

Modularity is a measure to evaluate the quality of community division within a network. Modularity can be interpreted as the degree to which the connections within a community are more organized than those between communities. If the connections within a community are significantly more numerous than those between communities, the modularity value will be relatively high (ref. Figure 1 below). Therefore, the problem of community detection can be transformed into a problem of maximizing modularity in a network.

### 1.4. The Numerical Simulation

To define the modularity function, first consider the adjacency matrix $A_{vw}$ where the element $A_{vw}=1$ indicates a connection between node *v* and node *w*; otherwise, $A_{vw}=0$. Let the sum of all elements in $A_{vw}$ be *m*, which represents the total number of edges (i.e., $m=\sum A_{vw}/2$). The basic idea behind modularity-based community detection is to maximize the number of edges within a community while minimizing the number of edges between communities. One assumes the network is divided into several communities and $C_{v}$ represents the community to which node *v* belongs. $\delta (C_{v},C_{w})=1$ if $C_{v}=C_{w}$ (i.e., node *v* and node *w* are in the same community) and $\delta (C_{v},C_{w})=0$ otherwise. Then, the modularity function *M* is defined as follows:

(1) $$M=\frac{1}{2m}\sum_{v,w}\left(A_{vw}-\frac{k_{v}k_{w}}{2m}\right)\delta \left(C_{v},C_{w}\right)$$

Here, the first term, $A_{vw}$, represents the actual number of edges between nodes *v* and *w*. The term $k_{v}$ is defined as the degree of the node, which is the number of edges connected to *v*:

(2) $$k_{v}=\sum_{w}A_{vw}$$

Therefore, $k_{v}k_{w}/2m$ is the expected number of edges between nodes *v* and *w* in a randomly connected network. The difference $A_{vw}-\frac{k_{v}k_{w}}{2m}$ reflects the deviation of the actual network from a random network. Thus, the modularity function *M* quantifies the extent to which the actual network structure is more modular (i.e., has more edges within communities) compared to a random network. In summary, modularity *M* can be used to determine the degree of community organization in a network.

To formulate the problem of maximizing modularity as a Quadratic Unconstrained Binary Optimization (QUBO) model, a binary decision variable $x_{vc}$ is defined, where $x_{vc}=1$ indicates that node *v* is assigned to community *c*; otherwise, it is 0. The modularity function *M* can then be expressed as

(3) $$M=\frac{1}{2m}\sum_{v,w,c}\left(A_{vw}-\frac{k_{v}k_{w}}{2m}\right)x_{vc}x_{wc}$$

Our goal is to maximize the modularity *M*. Since each node can only belong to one community, there is a constraint for any node. That is,

(4) $$\sum_{c}x_{vc}=1$$

Combining the above objective and constraint linearly, we arrive at the following QUBO formulation:

(5) $$\min_{x}-\frac{1}{2m}\sum_{c}\sum_{v}\sum_{w}\left(A_{vw}-\frac{k_{v}k_{w}}{2m}\right)x_{vc}x_{wc}+P\sum_{v}{\left(\sum_{c}x_{vc}-1\right)}^{2}$$

where *P* represents the penalty for violating the constraint, which is set to be sufficiently large to ensure the fulfillment of the constraint yet not too large to adversely affect the optimization of the objective. Note that the number of binary variables or qubits required equals the number of nodes multiplied by the number of communities.

Note that if there are only two communities, the above QUBO model can be simplified to the following form without constraint terms:

(6) $$\min_{x}-\frac{1}{2m}\sum_{v}\sum_{w}\left(A_{vw}-\frac{k_{v}k_{w}}{2m}\right)\left(x_{v}x_{w}+\left(1-x_{v}\right)\left(1-x_{w}\right)\right)$$

In this case, the number of binary variables or qubits needed is equal to the number of nodes instead of being twice the number of nodes.

### 1.5. The Equivalence Between the QUBO and Ising Models

A QUBO problem looks for a binary vector $x={\left(x_{1},x_{2},\ldots ,x_{n}\right)}^{T}$, where $x_{i}\in \left\{0,1\right\}$, to minimize the following objective function:

(7) $$f\left(x\right)=x^{T}Qx$$

where *Q* is a symmetric matrix of size n×n and $x^{T}$ is the transpose of *x*.

An Ising model is a physical model used to describe magnetic materials. It defines the energy of a set of spin variables $s=(s_{1},s_{2},\ldots ,s_{n})$, where $s_{i}\in \left\{-1,+1\right\}$. The energy function of their Ising model is expressed as follows:

(8) $$E\left(s\right)=-\sum_{i<j}J_{ij}s_{i}s_{j}-\sum_{i}h_{i}s_{i}$$

where $J_{ij}$ represents the interaction between spins $s_{i}$ and $s_{j}$ and $h_{i}$ is the external magnetic field acting on spin $s_{i}$. Although the QUBO model and the Ising model differ in form, they are mathematically equivalent. The following transformation can convert a QUBO problem into an Ising model and vice versa:

(9) $$x_{i}=\frac{1-s_{i}}{2}\;s_{i}=2x_{i}-1$$

where $x_{i}\in \left\{0,1\right\}$ is a binary variable in the QUBO model and $s_{i}\in \left\{-1,+1\right\}$ is the corresponding spin variable in the Ising model.

With this transformation, the energy function of the Ising model can be reformulated as the objective function of a QUBO problem. The mathematical equivalence dictates that when the energy of an Ising model reaches its minimum value, the objective function of the corresponding QUBO problem reaches its minimum and vice versa. This allows for solving QUBO problems through finding the ground state of the corresponding Ising models, which is particularly suited to using a Coherent Ising Machine (CIM). In our study, the transformation $s_{vc}=2x_{vc}-1$ is applied to Equation (5) above to arrive at the Ising model to be solved by CIM.

## 2. Materials and Methods

### 2.1. Graph Formation

The dataset used in this study comes from a Chinese commercial bank’s fraud detection scenario. The data collection process begins by randomly selecting cases of fraud from all detected fraudulent accounts. From these cases, all nodes with transactions (one-step connected) are extracted to form the first-degree association sample set. Next, for each sample in the first-degree association sample set, all nodes that have transactions (one-step connected) with these samples are identified, forming the second-degree association sample set. Finally, for each sample in the second-degree association sample set, all nodes that have transactions (one-step connected) with these samples are identified, forming the third-degree association sample set. The final sampled dataset contains a total of 3934 samples, among which 186 are labeled as fraudulent, accounting for approximately 5% of the total number of samples.

### 2.2. Data Preprocessing

Based on practical business experience, fraudulent transactions typically constitute a very small proportion of overall transaction volume. To obscure their fraudulent activities, fraudsters often generate a significant amount of transactional noise. This added noise further reduces the effectiveness of traditional detection methods, especially those relying on rule-based approaches. As a result, there is a compelling need to denoise transaction graph data. Denoising the transaction graph can significantly enhance the community detection capability of the model. Denoising also helps reduce the computational complexity by narrowing down the dataset to more pertinent interactions, making the model more efficient and effective in real time.

In this paper, we first employ rule-based methods to identify potential high-risk accounts. Next, we perform denoising on the graph data provided in the graph formation step by removing low-risk popular nodes and isolated nodes. With the above denoising procedure, we arrive at 308 accounts, 19 of which are fraudulent. Thus, the probability of fraud is 6.17% for the population. Although this denoising step may inevitably miss some fraudulent accounts, in practice the types of fraudulent entities vary widely—individual fraudsters, organized fraud networks and even occasional fraud cases. Hence, there is not an omnipotent approach for fraud detection and a combination of methods is required. Our community detection approach focuses on uncovering organized fraud networks, leaving individual or occasional fraud instances to be screened out by other rule-based or AI-based methods.

### 2.3. The CIM Setup

The CIM we used was provided by Beijing QBoson Quantum Technology Co. Ltd. (Beijing, China). This CIM consists of an optical part and an electrical part. The optical part of the CIM is composed of a pulsed laser, erbium-doped fiber amplifier (EDFA) fiber rings and periodically poled lithium niobate (PPLN) crystals, while the electrical part of the machine is composed of optical balanced homodyne detectors (BHDs), analog-to-digital/digital-to-analog (AD/DA) converters and field-programmable gate arrays (FPGAs).

During the operation, the 1560 nm pulsed laser emits a sequence of optical pulses, which are amplified by the EDFA, and then the frequency of the amplified laser pulses is doubled by a PPLN crystal to generate 780 nm laser pulses, which are used as the pump source to synchronously pump the phase-sensitive amplifier, producing degenerate optical parametric oscillation (DOPO). The output from the fiber ring cavity is measured through the BHDs, and the FPGA computes the feedback signal required to implement the optical coupling between laser pulses according to the interaction intensity between spins in the Ising Hamiltonian. This feedback signal is used as the control signal of the intensity modulator (IM) and the phase modulator (PM) during the next round trip [7,15].

The Louvain algorithm was run 100 times. Due to the non-deterministic nature of the algorithm, the community structure corresponding to the median value of the modularity function was used to represent the result of the algorithm. The simulated annealing (SA) algorithm was run 100 times as well, with an initial temperature of 10,000 degrees and a temperature decay rate of 0.99. In total, 1000 iterations were performed at each temperature. Again, the community structure corresponding to the median value of the modularity function was used to represent the result of the algorithm. We used a laptop equipped with a 12th Gen Intel Core i7-1255U CPU and 16 GB DDR4 memory to perform the classical simulation.

## 3. Result

As is mentioned above, the CIM utilizes FPGA to calculate the feedback signal, which requires digitization of the analog amplitude of laser pulses. Therefore, the QUBO matrix encoded by the FPGA has a precision limit. Here, we tried both the 8-bit and 14-bit QUBO matrix encoding precisions. We found that both managed to find the optimal community structure and their performance surpassed both the Louvain and SA algorithms (ref. Table 1 below).

As is shown in the comparison table, the CIM implementation of community detection has a better median value of modularity than the Louvain and SA algorithms. Although the median value of the 14-bit precision CIM is slightly lower than Louvain, the 8-bit precision CIM is far better, which can be attributed to being less prone to noise and precision error in the feedback signal. In addition, the time-to-solution of the CIM is at least one order of magnitude faster than that of the Louvain algorithm, and that of SA is much slower than both the CIM and Louvain. In terms of the success rate, the 8-bit precision CIM produced the highest modularity about 1/3 of the times, followed by 15% of the 14-bit precision CIM. In contrast, Louvain managed to reach the best modularity only once among the 100 trials, and neither the 8-bit SA nor the 14-bit SA was able to produce the best modularity. Hence, the CIM not only detected the community structure faster but also delivered better-quality results.

In terms of the community structure, the optimal structures (i.e., that with the highest modularity) achieved by the three are almost the same, and those of Louvain and the CIM are identical (ref. Table 2, Table 3 and Table 4 below). The community structure provides significant value to the commercial bank in identifying fraudulent accounts. The high-risk community stands out with a fraud probability of 14.4%, more than double that of the overall population (6.17%), and significantly higher than that of other communities. Furthermore, this high-risk community contains nearly 70% of all fraudulent accounts. This high recall rate will greatly improve the effectiveness of the bank’s fraud detection business.

## 4. Discussion

As is discussed in the QUBO model for community detection above, the number of binary variables or qubits required to implement community detection is equal to the number of nodes multiplied by the number of communities. Consequently, the number of qubits needed increases rapidly with the number of communities even if the number of nodes is fixed. This imposes limitations on the complexity of the community structure that can be analyzed using this method. If most fraudulent accounts operate individually, the corresponding transaction network may not be well suited for QUBO-based community detection implemented via quantum computing. Fortunately, in practice, many rule-based methods are available to pre-filter these individual fraudulent accounts, leaving behind organized crime units that are more suitable for community-based analysis.

On the other hand, real transaction networks in commercial banks are far more complex than the one analyzed in this study, often involving tens of millions of accounts. This level of complexity exceeds the capacity of algorithms like Louvain for real-time processing. Fortunately, most accounts in a transaction network are normal with stable interactions. As a result, community detection can be applied selectively, focusing only on the relevant parts of the network. This selective approach is another key advantage of the QUBO formulation over the Louvain method. With this technique and the rapid scaling of qubits in the CIM, we anticipate that quantum computing for community detection could be practically implemented in commercial banks’ anti-fraud business in the near future.

## 5. Conclusions

While there are many proposals of quantum algorithms for fraud detection, the hardware challenge of this application of quantum computing has not been addressed. In this paper, we utilized the Coherent Ising Machine (CIM) to perform community detection by framing the problem as a Quadratic Unconstrained Binary Optimization (QUBO) model to maximize the modularity of the resulting community structure. We compared our results with the classical Louvain method and simulated annealing (SA) algorithms. Our findings show that the CIM + QUBO approach not only completed the detection faster (∼1 ms) than both Louvain (∼10 ms) and SA (∼10^5^ ms) but also had a higher likelihood of producing the optimal solution (>10% vs. 1% for Louvain and 0% for SA). Additionally, the resulting community structure holds substantial business value, particularly in the clear identification of high-risk communities and its coverage of fraudulent accounts (≈70%). It highlights the great potential of quantum computing in solving complex real-world problems, especially in the financial sector. This study also expands the application of the Ising machine, extending its use beyond federated learning [25], incremental learning [26] and quantum annealing [27]. These AI-based methods, once implemented by the CIM, could lead to more applications of quantum computing for fraud detection in the future.

## Figures and Tables

**Table 1:** Community detection performance comparison among the classical Louvain and SA algorithms and the CIM, where the success rate is defined as the proportion of the optimal modularity results.

	Louvain	SA (14-bit)	SA (8-bit)	CIM (14-bit)	CIM (8-bit)
modularity (median)	0.7062	0.5898	0.5253	0.6828	0.7088
modularity (max)	0.7089	0.7038	0.6967	0.7089	0.7089
time-to-solution in milliseconds (median)	35.474	2,414,001.600	269,003.000	1.860	0.263
time-to-solution in milliseconds (min)	24.517	104,872.000	259,830.332	0.367	0.093
success rate	1.0%	0.0%	0.0%	15.0%	32.0%
fraud probability of the high-risk community	14.4%	14.0%	14.3%	14.4%	14.4%
recall rate of fraud accounts	68.4%	63.2%	68.4%	68.4%	68.4%

**Table 2:** The optimal community structure produced by the CIM.

ID	Node Count	Fraudulent Accounts	Fraud Probability	Recall Rate
0	61	1	0.016393	0.052632
1	80	5	0.062500	0.263158
2	77	0	0.000000	0.000000
3	90	13	0.144444	0.684211
Total	308	19	0.061688	1.000000

**Table 3:** The optimal community structure produced by the Louvain algorithm.

ID	Node Count	Fraudulent Accounts	Fraud Probability	Recall Rate
0	61	1	0.016393	0.052632
1	90	13	0.144444	0.684211
2	80	5	0.062500	0.263158
3	77	0	0.000000	0.000000
Total	308	19	0.061688	1.000000

**Table 4:** The optimal community structure produced by the SA algorithm.

ID	Node Count	Fraudulent Accounts	Fraud Probability	Recall Rate
0	76	4	0.052632	0.210526
1	78	1	0.012821	0.052632
2	91	13	0.142857	0.684211
3	63	1	0.015873	0.052632
Total	308	19	0.061688	1.000000

## Data Availability

The data presented in this study are available on request from the corresponding author.
